# VEGFR2 alteration in Alzheimer’s disease

**DOI:** 10.1038/s41598-017-18042-1

**Published:** 2017-12-18

**Authors:** Sun-Jung Cho, Moon Ho Park, Changsu Han, Keejung Yoon, Young Ho Koh

**Affiliations:** 1 Division of Brain Diseases, Center for Biomedical Sciences, Korea National Institute of Health, 187 Osongsaengmyeong2-ro, Osong-eup, Heungdeok-gu, Cheongju-si, Chungcheongbuk-do 28159 Korea; 2 Departments of Neurology, Korea University Medical College, Ansan Hospital, 123 Jeokgeum-ro, Danwon-gu, Ansan-si, Gyeonggi-do 15355 Korea; 3 Psychiatry, Korea University Medical College, Ansan Hospital, 123 Jeokgeum-ro, Danwon-gu, Ansan-si, Gyeonggi-do 15355 Korea; 4 College of Biotechnology and Bioengineering, Sungkyunkwan University, 2066 Seobu-ro, Jangan-gu, Suwon-si, Gyeonggi-do 16419 Korea

## Abstract

Alzheimer’s disease (AD) is a common disorder of progressive cognitive decline among elderly subjects. Angiogenesis-related factors including vascular endothelial growth factor (VEGF) might be involved in the pathogenesis of AD. Soluble form of the VEGF receptor is likely to be an intrinsic negative counterpart of VEGF. We measured the plasma levels of VEGF and its two soluble receptors (sVEGFR1 and sVEGFR2) in 120 control subjects, 75 patients with mild cognitive impairment, and 76 patients with AD using ELISA. Plasma levels of VEGF in patients with AD were higher than those in healthy control subjects. However, plasma levels of sVEGFR1 and sVEGFR2 were lower in patients with AD than in healthy control subjects. Levels of *VEGFR2* mRNA were significantly decreased in human umbilical vein endothelial cells after amyloid-beta treatment. Further, protein levels of VEGFR2 were also decreased in the brains of AD model mice. In addition, we show that the expression of sVEGFR2 and VEGFR2 was also decreased by the transfection with the Notch intracellular domain. These results indicate that the alterations of VEGF and its two receptors levels might be associated with those at risk for Alzheimer’s disease.

## Introduction

Alzheimer’s disease (AD) is characterized by the progressive loss of cognitive function leading to dementia and by the accumulation of amyloid-beta (Aβ) in the brain^1^. Aβ peptides are also considered to cause microvascular degeneration, cerebral amyloid angiopathy (CAA), vessel wall rupture, and cerebral perfusion deficits^2^. Reactive angiogenesis may be induced by cerebral ischemia and by the upregulation of vascular endothelial growth factor (VEGF)^3^. In spite of increases in angiogenic factors in AD, abnormalities in cerebrovasculature have often been reported in AD and CAA. Several studies have been shown the role of Aβ as an inhibitor of angiogenesis^4–6^.

The VEGF signal cascade is known to activate angiogenic, neurotrophic, and cytoprotective processes^7^. VEGF plays diverse roles within the brain and promotes neural cell survival^8^. Neuronal survival effects of VEGF in brain pathologies, such as stroke and Parkinson’s disease (PD), have been reported^9,10^. Deposition of Aβ peptides is suggested to increase inflammatory cytokines such as TNFα in the AD brain^11^. Expressions of VEGF and VEGFR1 was increased in the microglia of brain tissue of patients with AD and in Aβ-treated microglia, indicating a role of VEGFR1 as a microglial chemotactic receptor^12^. Therefore, VEGF and VEGFR are therapeutic targets for various brain diseases.

During the angiogenesis process, the most potent mitogens acting on endothelial cells (EC) are the VEGF. The stimulation of a type 2 receptor specific for VEGF (VEGFR2, or fetal liver kinase-1 (FLK-1), or kinase insert domain receptor (KDR)) activates endothelial nitric oxide synthase (eNOS). This enhances the release of nitric oxide that extends and increases the permeability of the vessel, which is vital for the start of angiogenesis. VEGFR1 and VEGFR2 occur in two isoforms, a full-length form (VEGFR1 and VEGFR2) and a shortened soluble form (sVEGFR1 and sVEGFR2). Both sVEGFR1 and sVEGFR2 are the products of alternative mRNA splicing. Since sVEGFR1 and sVEGFR2 have only extracellular immunoglobulin-like domains, they can be released into the blood and exerts reducing effects on VEGF signaling by playing the role of a ligand-trap^13,14^.

Recent studies have shown an increase in VEGF in the cerebrospinal fluid (CSF) and peripheral blood in patients with AD^15^. Expression levels of *VEGF* mRNA and *VEGFR2* mRNA were increased in the entorhinal cortex of a mouse model of AD^16^. Since soluble VEGFR can act as a natural VEGF inhibitor, it might be important to understand the correlation of VEGF and VEGFR levels in AD. At present, the clinical significance of increased plasma VEGF levels is well studied in patients with dementia. Recently, it has been previously reported that the serum levels of VEGF are lower in AD. Alteration of VEGF levels in AD is controversial. However, that of plasma levels of soluble VEGFR as a natural VEGF inhibitor is still unknown.

VEGFR2-mediated cellular metabolic activity has been reported to be associated with lipid rafts and caveolae/raft-dependent endocytosis, which can also modulate VEGF signal transduction cascades^17,18^. It was also reported that the alteration of peripheral blood lipids are associated with AD^19^. We analyzed the correlations between plasma VEGF and VEGFRs levels and lipid parameters that might be involved in dysregulated lipid conditions in AD.

In this study, we have evaluated whether there are changes in VEGFR levels in AD and determined the role of Notch-1 on the regulation of VEGFR. We demonstrated that sVEGFR1 and sVEGFR2 levels are significantly decreased in the plasma of patients with AD. We further found that upregulation of Notch-1 might be involved in VEGFR regulation. These results highlight the importance of sVEGFR as a potent biomarker for AD.

## Results

### Analysis of correlations between plasma VEGF, sVEGFR1 and sVEGFR2 levels

Table 1 presents participant characteristics. Patients with dementia were older compared to control subjects. The mean age of the normal control participants was 71.9 ± 0.42 years, and the majority of normal control participants were women (58%). The mean age of the dementia participants was 75.1 ± 0.6 years, and the majority of them were women (75%). The subjects with mild cognitive impairment (MCI) had a mean age of 73.01 ± 0.51 years, and 60% of them were women. Patients with dementia were less educated than normal controls. The overall MMSE score was lower in patients with dementia, while it was in the normal range in MCI and healthy controls.

**Table 1:** Baseline characteristics of the population.

Features	Control	MCI	Dementia	*p*-value
N (Male/Female)	120 (50/70)	75 (29/ 46)	76 (18/ 58)	
Age (yr)	71.9 ± 0.42	73.01 ± 0.51	75.1 ± 0.6	<0.001
MMSE	27.24 ± 0.19	24.83 ± 0.35	16.09 ± 0.66	<0.001
CDR	0.04 ± 0.01	0.27 ± 0.02	1.15 ± 0.08	<0.001
Total CHOL	195.8 ± 3.1	190.9 ± 4.1	203.1 ± 4.0	0.161
TG	137.3 ± 6.9	132.4 ± 7.6	161.4 ± 11.2	0.09
HDL	43.4 ± 0.8	42.8 ± 1.4	44.1 ± 1.1	0.343
LDL	124.9 ± 2.9	121.6 ± 3.5	126.7 ± 3.7	0.511
ApoE ε4 allele, N(%)				
Absent (−)	98 (80%)	66 (88%)	50 (65.8%)	
Present (+)	24 (20%)	9 (12%)	26 (34.2%)	

Values are mean ± SEM.

Key: MCI, mild cognitive impairment; MMSE, Mini-Mental State Examination; CDR, clinical dementia rating; SEM, standard error of the mean.

For the analysis of VEGF, sVEGFR1, and sVEGFR2 concentrations in plasma, we measured their plasma protein levels using commercial ELISA kits. Plasma concentrations of sVEGFR1 were different between the three groups (*p* < 0.005; Kruskal-Wallis test) (Table 2). A decrease in sVEGFR1 concentrations was observed in subjects with dementia (107 ± 1.8 pg/mL), as compared to the control subjects (119 ± 4.5 pg/mL) (*p* = 0.008; Mann-Whitney U-tests). Plasma concentrations of sVEGFR2 were different between the three groups (*p* = 0.046; Kruskal-Wallis test) (Table 2). A decrease in sVEGFR2 concentrations was observed in subjects with dementia (6.89 ± 0.13 ng/mL), as compared to the control subjects (7.39 ± 0.12 ng/mL) (*p* = 0.011; Mann-Whitney U-tests).

**Table 2:** Analysis of sVEGFR1 and sVEGFR2 concentrations in plasma.

Features	Control	MCI	Dementia	*p*-value
N (Male/Female)	120 (50/70)	75 (29/ 46)	76 (18/ 58)	
sVEGFR1 (pg/ml)	119 ± 4.5	116 ± 3.6	107 ± 1.8^a^	**<0.005**
sVEGFR2 (ng/ml)	7.39 ± 0.12	7.18 ± 0.13	6.89 ± 0.13^b^	**<0.05**

Values are mean ± SEM.

^a^Compared with control; *p* = 0.008, ^b^Compared with control; *p* = 0.011.

Plasma concentrations of VEGF, VEGF/sVEGFR1, and VEGF/sVEGFR2 were different between the three groups (*p* < 0.05; Kruskal-Wallis test) (Table 3). Plasma concentrations of VEGF in subjects with dementia (167 ± 11.8 pg/mL) were higher than in control subjects (*p* = 0.013; Mann-Whitney U-tests). VEGF levels were 17% higher in plasma samples of dementia patients compared to normal controls. Furthermore, VEGF/sVEGFR1 levels were 27% higher and VEGF/sVEGFR2 levels were 29% higher in plasma samples of dementia patients compared to normal controls (*p* = 0.004 and *p* = 0.002, respectively) (Table 3). VEGF/sVEGFR1 levels were 34% higher and VEGF/sVEGFR2 levels were 36% higher in plasma samples of MCI subjects compared to normal controls (*p* = 0.019 and *p* = 0.012, respectively) (Table 3).

**Table 3:** Analysis of VEGF, VEGF/sVEGFR1 and VEGF/sVEGFR2 in plasma.

Features	Control	MCI	Dementia	*p*-value
N (Male/Female)	120 (50/70)	75 (29/46)	76 (18/58)	
VEGF-A (pg/ml)	142 ± 7.4	185 ± 21	167 ± 11.8^a^	**<0.05**
VEGF/sVEGFR1	1.27 ± 0.06	1.71 ± 0.21	1.62 ± 0.12^b^	**<0.05**
VEGF/sVEGFR2	19.63 ± 1.0	26.8 ± 3.2	25.2 ± 1.8^c^	**<0.01**

Values are mean ± SEM.

Correlations between two groups (Control and Dementia) are shown as below.

^a^Compared with control; *p* = 0.013.

^b^Compared with control; *p* = 0.004.

^c^Compared with control; *p* = 0.002.

**Figure 1:**
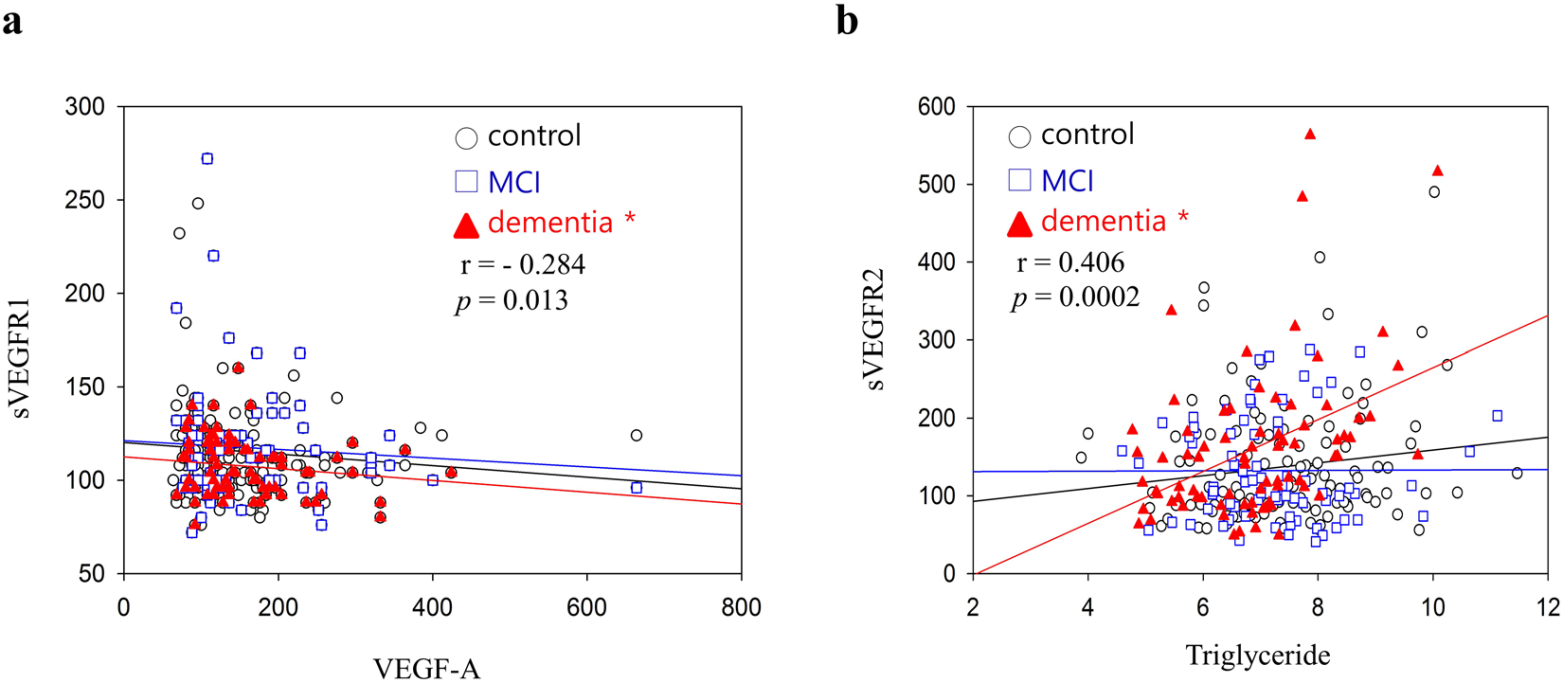
Analysis of sVEGFR1 and sVEGFR2 concentrations in human plasma. Plasma sVEGFR1 and sVEGFR2 concentrations were measured by ELISA. The differences in the relative amounts of sVEGFR1 and sVEGFR2 were compared between dementia, MCI, and healthy controls by means of Mann-Whitney’s U-test within different groups. (**a**) Plasma sVEGFR1 levels in dementia subgroup were not closely correlated with VEGF-A (*r* = −0.284, *p* = 0.013) (**b**) Plasma sVEGFR2 levels in dementia subgroup were significantly correlated with triglyceride (*r* = 0.406, *p* = 0.0002).

Supplementary Table 1 shows that plasma levels of sVEGFR1 were correlated with VEGF and sVEGFR2 levels (*r* = −0.156, *p* = 0.01 and *r* = 0.154, *p* = 0.011, respectively). Within the dementia subgroup, levels of sVEGFR1 were not closely correlated with VEGF (*r* = −0.284, *p* = 0.013) (Fig. 1a). There was no association between VEGF and sVEGFR2 levels. The results of the MMSE assessments were significantly correlated with sVEGFR1, VEGF, VEGF/sVEGFR1, and VEGF/sVEGFR2 levels. The results of the CDR assessment were significantly correlated with sVEGFR2 and VEGF/sVEGFR2 levels.

### Analysis of correlations between plasma VEGF and sVEGFR levels and lipid parameters

Subsequently, we examined the association between plasma sVEGFR and VEGF levels and lipid parameters. Supplementary Table 1 shows the relationship between VEGF, its antagonists (sVEGFR1 and sVEGFR2), and lipid parameters. Levels of sVEGFR2 were significantly correlated with triglyceride (TG) in all subjects (*r* = 0.13, *p* = 0.03) (Supplementary Table 1). Within the dementia subgroup, levels of sVEGFR2 were significantly correlated with TG (*r* = 0.406, *p* = 0.0002) (Supplementary Table 2 and Fig. 1b). Levels of sVEGFR1 were closely correlated with total cholesterol (TC) and low-density lipoprotein (LDL) (*r* = −0.187, *p* = 0.002 and *r* = −0.164, *p* = 0.007, respectively) (Supplementary Table 1). Levels of sVEGFR1 in the MCI subgroup were correlated with TC and LDL (*r* = −0.232, *p* = 0.045 and *r* = −0.259, *p* = 0.025, respectively) (Supplementary Table 2).

Under conditions of high levels of LDL (>130 mg/dl), sVEGFR2 levels were 10% lower in the plasma of dementia patients compared to normal controls (*p* = 0.013) (Supplementary Table 3). VEGF, VEGF/sVEGFR2, and VEGF/sVEGFR1 levels were 35%, 50%, and 42% higher in plasma samples of dementia patients compared to normal controls (*p* = 0.019, *p* = 0.003 and *p* = 0.025, respectively). Under conditions of high levels of TC (>220 mg/dl), VEGF, VEGF/sVEGFR2, and VEGF/sVEGFR1 levels were 71%, 90% and 84% higher in the plasma of dementia patients compared to normal controls (*p* = 0.002, *p* = 0.0001 and *p* = 0.0001, respectively) (Supplementary Table 4). For ApoE ε4 carriers, sVEGFR2 levels were significantly decreased in patients with dementia compared to normal controls (*p* = 0.009; Mann-Whitney U-tests) (Supplementary Table 5). For dementia patients, sVEGFR2 levels were 7% lower in ApoE ε4 carriers than in non-carriers, but the difference was not significant (*p* = 0.073).

We compared the area under the ROC curve (AUC) for all analysis measures derived from the plasma samples of the patients (Table 4). The accuracy of the ROC curve on dementia versus control using plasma VEGF levels was 0.605 (*p* < 0.05). Using the ratio of VEGF to sVEGFR (VEGF/sVEGFR2 and VEGF/sVEGFR1) improved accuracy 0.629 and 0.623, respectively. To combine these biomarkers, we tested a logistic regression model and found that, as illustrated in Fig. 1, this combination resulted in an important improvement, with accuracy reaching 0.736.

**Table 4:** ROC curve (AUC) of all the analyses with VEGF, sVEGFR1, and sVEGFR2 measured in the plasma.

Features	AUC	95% CI	*p*-value
VEGF	0.605	0.524–0.686	0.013
VEGF/sVEGFR1	0.623	0.542–0.702	0.0038
VEGF/sVEGFR2	0.629	0.549–0.708	0.00235
sVEGFR1	0.608	0.527–0.688	0.010
sVEGFR2	0.613	0.530–0.693	0.0077
sVEGFR1 + sVEGFR2	0.643	0.566–0.721	0.001
VEGF + VEGF/sR1 + VEGF/sR2	0.662	0.585–0.740	<0.001
Age + APOE + VEGF + VEGF/sR1 + VEGF/sR2	0.736	0.662–0.811	<0.001

### sVEGFR levels were modulated by Aβ *in vitro*

**Figure 2:**
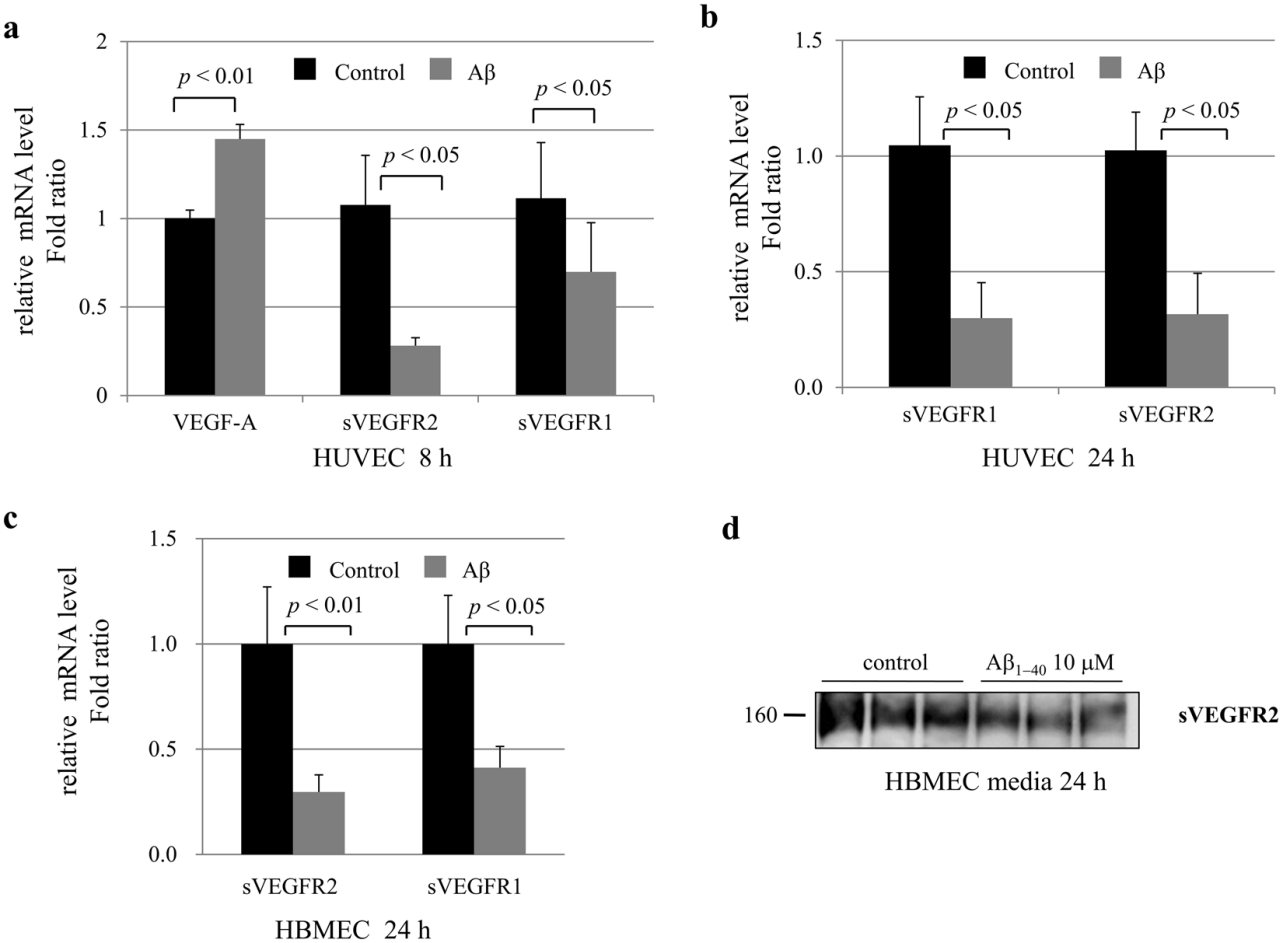
*sVEGFR2* and *sVEGFR1* mRNA expression in endothelial cells. (**a**) HUVEC were treated with 10 μM of Aβ_1–40_ peptides for 8 h. Real-time PCR results showing relative mRNA expression levels of *VEGF-A*, *sVEGFR2*, and *sVEGFR1* (n = 3). (**b**) After HUVEC were treated with 10 μM of Aβ_1–40_ peptides for 24 h, sVEGFR1 and *sVEGFR2* mRNA expression levels were measured by real-time PCR (n = 3). (**c**) HBMEC were treated with 10 μM of Aβ_1–40_ peptides for 24 h. *sVEGFR2* and *sVEGFR1* mRNA expression levels were measured by real-time PCR (n = 3). (**d**) Splicing variant form of VEGFR2 (sVEGFR2) protein levels were detected in HBMEC cell culture media. The cropped blot is displayed in the main figure, and its full-length blot is presented in Supplementary Figure 1. Treatment of 10 μM of Aβ_1–40_ peptides for 24 h decreased the sVEGFR2 levels (n = 3).

To explore molecular mechanisms underlying sVEGFR level alterations in AD, we investigated sVEGFR levels in endothelial cells (EC). We examined whether Aβ could induce a decrease in sVEGFR, possibly explaining level alterations observed in human subjects. Although Aβ peptides increases *VEGF* mRNA expression in the brain, the corresponding alteration of sVEGFR levels in EC is unknown. After EC were treated with Aβ_1–40_ peptides for 8 h or 24 h, mRNA expression levels of target genes were measured by real-time PCR. The levels of *VEGF* mRNA were significantly increased in human umbilical vein endothelial cells (HUVEC) after Aβ treatment for 8 h (Fig. 2a). The levels of *sVEGFR2* mRNA were significantly decreased in HUVEC after Aβ treatment for 8 h or 24 h (Fig. 2a and b). The levels of *sVEGFR1* mRNA were significantly decreased in HUVEC after Aβ treatment for 24 h (Fig. 2b). We also found that mRNA levels of *sVEGFR2* were significantly decreased after treatment with 10 μM Aβ_1–40_ peptides for 24 h in human brain microvascular endothelial cells (HBMEC) (Fig. 2c). Using a specific antibody to the splice variant form of VEGFR2 (sVEGFR2), we then examined the sVEGFR2 protein levels in cell culture media. Treatment with Aβ decreased sVEGFR2 levels in HBMEC culture media (Fig. 2d). These findings suggest that Aβ may inhibit *sVEGFR2* mRNA levels in EC and lead to enhanced VEGF reactivity by decreasing plasma levels of sVEGFR2.

### VEGFR2 and sVEGFR2 levels were decreased by Aβ

**Figure 3:**
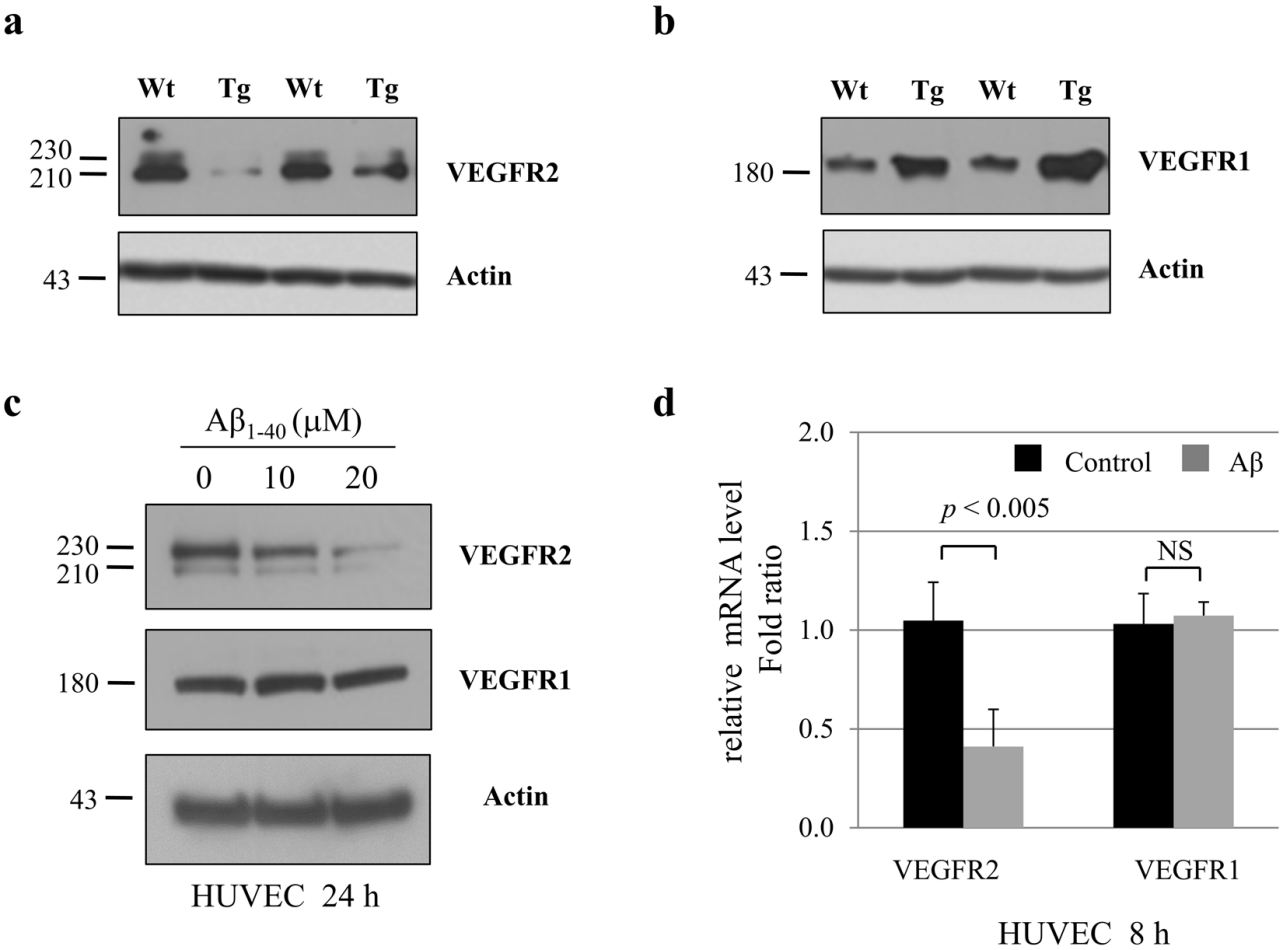
VEGFR2 and VEGFR1 expression in endothelial cells. VEGFR2 (**a**) and VEGFR1 (**b**) expressions in the brain of 18-month-old wild type and APPsw/PS1ΔE9 transgenic mice were determined by immunoblot assay. The cropped blots are displayed in the main figures, and its full-length blots are presented in Supplementary Figure 2a and b. Actin was used as a loading control. (**c**) After HUVEC were treated with 10 μM or 20 μM of Aβ_1–40_ peptides for 24 h, VEGFR2 and VEGFR1 protein levels were measured by immunoblot assay. The cropped blots are displayed in the main figures, and its full-length blots are presented in Supplementary Figure 2c. Actin was used as a loading control. (**d**) HUVEC were treated with 10 μM of Aβ_1–40_ peptides for 8 h. Real-time PCR results showing relative mRNA expression levels of *VEGFR2* and *VEGFR1* (n = 3). NS = not significant.

We next investigated the levels of VEGF receptors in the APPsw/PS1ΔE9 transgenic (Tg) mouse brain. Western blotting analysis was performed using cortex homogenates of 18-month-old wild-type and APPsw/PS1ΔE9 Tg mice. Levels of VEGFR2 were markedly decreased in the APPsw/PS1ΔE9 Tg mouse cortex compared with wild-type controls (Fig. 3a). However, we found that the levels of VEGFR1 increased in the APPsw/PS1ΔE9 Tg mouse cortex compared with wild-type controls (Fig. 3b). Furthermore, we examined whether VEGFR2 levels are decreased in Aβ-treated EC. After cells were treated with Aβ, we found that Aβ induces a decrease in VEGFR2 of both protein (Fig. 3c) and mRNA (Fig. 3d) levels. There was no change in *VEGFR1* mRNA levels (Fig. 3d) and VEGFR1 protein levels after Aβ treatment for 24 h (Fig. 3c).

**Figure 4:**
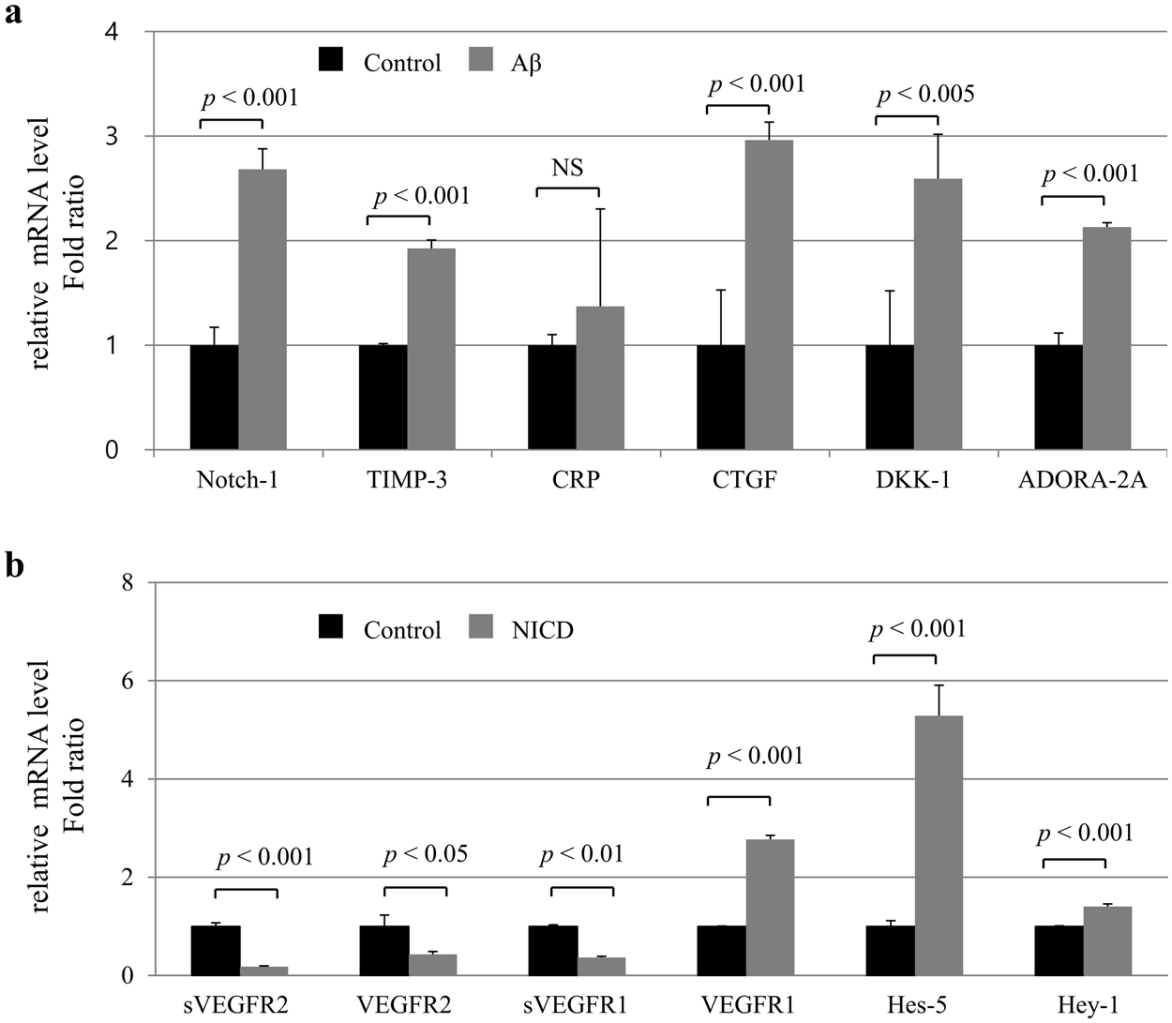
*Notch-1* and *VEGFR2* mRNA expression in endothelial cells. (**a**) HUVEC were treated with 10 μM of Aβ_1–40_ peptides for 8 h. Real-time PCR results showing relative mRNA expression levels of *Notch-1*, *TIMP-3*, *CRP*, *CTGF*, *DKK-1*, and *ADORA-2A* (n = 3). (**b**) HUVEC were transiently transfected with the Notch intracellular domain (NICD) for 48 h. *sVEGFR2*, *VEGFR2*, *sVEGFR1*, *VEGFR1*, *Hes-5*, and *Hey-1* mRNA expression levels were measured by real-time PCR (n = 3). NS = not significant.

It has been reported that VEGFR2 signaling is downregulated by Notch-1^20^, CRP^21^, and tissue inhibitor of metalloproteinases-3 (TIMP-3)^22^. To investigate molecular mechanisms for Aβ-mediated VEGFR2 downregulation, we next examined the alterations in gene expression of *NOTCH-1*, *CRP*, and *TIMP-3*. After HUVEC were treated with 10 μM of Aβ_1–40_ peptides for 8 h, we found that the mRNA levels of *NOTCH-1* and *TIMP-3* had significantly increased (Fig. 4a).

To investigate molecular mechanisms for Aβ-mediated VEGFR2 downregulation, we then examined whether Notch signaling is involved in VEGFR2 downregulation. HUVEC were transiently transfected with the control GFP (GIA), GFP-linked Notch intracellular domain (GNIA) cloned into the IRES-eGFP vector. After 48 h of incubation, fluorescent images of GFP expressions were shown in Supplementary Figure 3. Further, the mRNA levels of *sVEGFR2* and *VEGFR2* were significantly decreased by transfection with the Notch intracellular domain (NICD) (Fig. 4b). Transfection of NICD increased *VEGFR1* gene expression, but decreased *sVEGFR1* gene expression. As we expected, transfection of NICD increased gene expression levels of *Hes-5* and *Hey-1*, which are the target genes of NICD.

**Figure 5:**
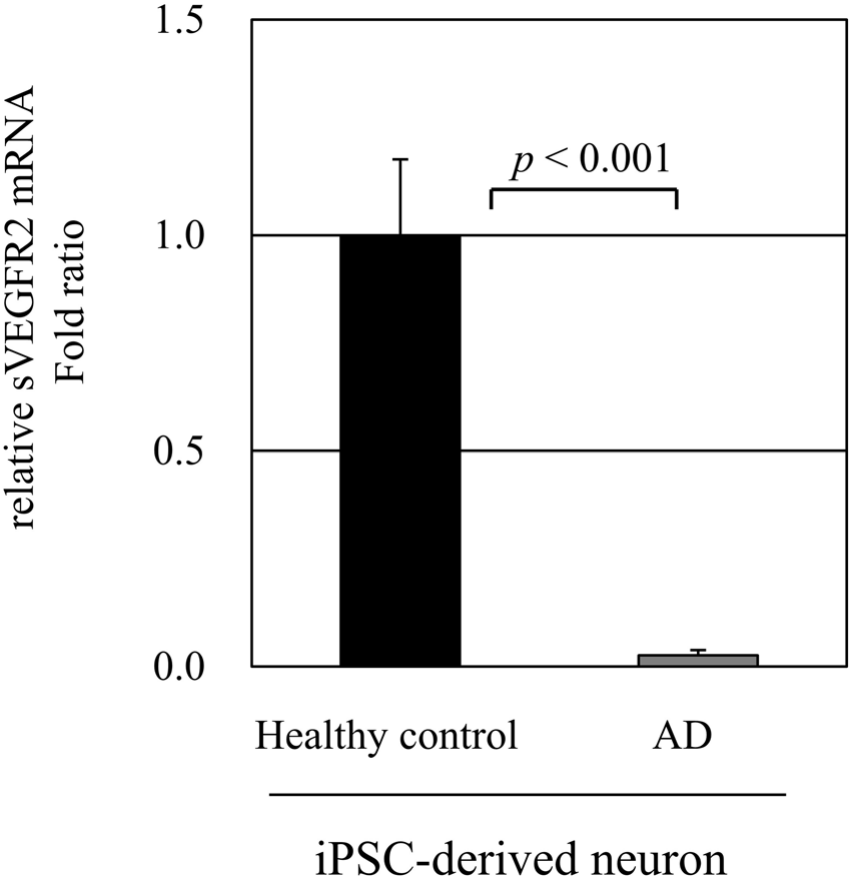
*sVEGFR2* mRNA expression in human iPSC-derived neuronal cells. Relative *sVEGFR2* mRNA expression levels were measured on human iPSC-derived neural progenitor stem cells from AD patient and healthy control (n = 3). Human iPSC cells were differentiated to neurons with treatment of neuronal differentiation media. *sVEGFR2* mRNA expression was significantly decreased in human iPSC cells from AD patient (*p* < 0.001).

We next examined sVEGFR2 levels in human iPSC-derived neural progenitor stem cells from patient with AD. After cells were differentiated to neurons, the mRNA levels of *sVEGFR2* were decreased in patient with AD compared to healthy control (Fig. 5). These results suggest that sVEGFR2 levels were decreased in patients with AD.

## Discussion

The present study demonstrates that plasma levels of sVEGFR1 or sVEGFR2 correlate with cognitive decline in patients with dementia. This study is the first to address plasma levels of sVEGFR1 or sVEGFR2, as well as VEGF and their clinical significance in AD. We found that plasma levels of sVEGFR1 or sVEGFR2 were significantly lower in patients with AD than in patients with MCI or healthy control subjects. VEGF, VEGF/sVEGFR2, and VEGF/sVEGFR1 levels were higher in patients with dementia compared to healthy control subjects (17%, 29% and 27%, respectively).

Expression of sVEGFR has been studied as a non-invasive biological markers. The alternative splice variant form of sVEGFR2 has been reported recently^23^ and plays a role as an endogenous VEGF-C antagonist. Levels of sVEGFR2 were higher in patients with systemic sclerosis^24^. It has also been reported that plasma sVEGFR2 levels were found to be decreased during pregnancies with intrauterine growth retardation^25^. Lamszus *et al*. found that the ratio of sVEGFR1 to VEGF is decreased in glioblastoma^26^. Considering the role of Aβ as an inhibitor of angiogenesis^5^, it is important to understand the clinical implication of sVEGFR as a VEGF antagonist in AD. This study shows that plasma levels of sVEGFR2 and sVEGFR1 are lower in patients with dementia than in cognitively healthy subjects. Given that VEGF is a potent inducer of vascular permeability at the blood-brain-barrier (BBB)^27^, the possibility that an increase in VEGF could attenuate sVEGFR levels is important to consider for the pathology of AD including BBB leakage. VEGF levels are increased in tissues obtained from patients with AD^12^ and VEGF decreases blood sVEGFR2 levels as a result of a ligand-mediated decrease in VEGFR2^28^. Experiments conducted *in vitro* show that these results may be explained by Aβ-mediated VEGF upregulation, which in turn could leads to reduced sVEGFR levels^28^, thus implicating that expression levels of sVEGFR2 in endothelial cells may explain modulation of plasma sVEGFR2 levels in patients with AD.

Several studies have suggested that there is a close relationship between VEGF and sVEGFR. VEGF and sVEGFR2 in the blood were found to be inversely correlated in various diseases including lymphoblastic leukemia and placenta accreta^29,30^. Our study shows that the ratio of VEGF to sVEGFR was significantly increased in patients with AD. Levels of sVEGFR1 in the dementia subgroup were significantly and negatively correlated with VEGF (*r* = −0.284, *p* = 0.013). Although an aspect of our finding with VEGF and sVEGFR2 may also offer insight into the similar inverse trend, we found no correlation between VEGF and sVEGFR2.

Angiogenesis and vascular dysfunction may be involved in neurodegeneration^31^. Although AD is a multifactorial disease, recent observations indicate that pro-angiogenic factors are related to the pathogenesis of the disease. Given that the release of VEGF in AD may contribute to angiogenesis, angiogenesis might be involved in Aβ accumulation in AD^32^. Previous study showing VEGF-mediated tight junction dysregulation implicates that VEGF may contribute to the dysregulation of the BBB^33^. VEGF levels are increased in the CSF and blood of patients with AD with a correlation of those levels with the clinical severity of AD^34–36^. Our results support these findings, as we detect that blood VEGF levels are increased in patients with AD. Since sVEGFR acts as an endogenous VEGF antagonist, the ratio of VEGF to sVEGFR is also noteworthy. VEGF induces VEGFR2 endocytosis and leads to the downregulation of VEGFR2 levels on the endothelial cell surface^37^. In addition, VEGF has been shown to decrease sVEGFR2 levels *in vitro* ^28^. The present study shows that the VEGF/sVEGFR ratio was increased in patients with AD, implicating elevated free VEGF levels in the plasma to bind membrane-anchored VEGF receptors. Our study also shows that the ratio of VEGF to sVEGFR1 is significantly altered under conditions of low levels of HDL. Likewise, under conditions of high levels of LDL, the ratio of VEGF to sVEGFR2 is significantly altered as well.

In endothelial cells (EC), VEGFR1 could play the role of a sink to trap an excess of VEGF because of its high affinity to VEGF, which is ten-fold higher than that of VEGFR2, while VEGFR2 is regarded to be the major signaling receptor. A reduction of sVEGFR in the blood may lead to enhanced VEGF reactivity around vessels, promoting active angiogenesis and vascular permeability. Previous study has also reported upregulation of VEGFR1 in patients with AD^12^. In mouse model of AD, we also observed that VEGFR1 levels were increased in the mouse brain, while VEGFR2 levels were decreased. Although VEGF levels are increased in AD, increased VEGFR1 expression might serve as a trap for free VEGF to suppress the pro-angiogenic function of VEGFR2^38^. Previous studies have reported that Aβ causes anti-angiogenesis effects via FGF-2 production^6,39^. It is thus noteworthy that VEGFR2 levels were decreased in the APPsw/PS1ΔE9 transgenic (Tg) mouse brain in the current study. Given that decreased VEGFR2 levels in diabetes lead to an impairment in angiogenesis^40^, downregulated VEGFR2 levels in Aβ-treated endothelial cells, and, as a consequence, a suppressed signal response of VEGFR2, may provide an explanation for impaired angiogenesis in AD. VEGFR2 levels are significantly decreased in Aβ-treated endothelial cells which might be linked to dysregulation of VEGF signaling. Further studies will be required to examine Aβ and VEGF levels in AD.

Notch signaling in EC has been proposed to modulate angiogenesis through the repression of VEGFR2^20^. The cytoplasmic domain of Notch (NICD) is proteolytically cleaved by γ-secretase and then translocates to the nucleus for upregulation of proteins like Hey-1 and Hes-5^41^. Previous studies have reported an upregulation of Notch-1 in the AD brain^42,43^. Consistent with these findings, in the current study, levels of Notch-1 expression in the EC were increased, and the downregulated VEGFR2 levels were also observed in Aβ-treated EC and in the APPsw/PS1ΔE9 Tg mouse brain. Given that Notch-1 is upregulated by VEGF^44^, elevation of plasma VEGF levels might be associated with Notch-1 expression in EC.

Dysregulated levels of lipid including LDL have been recognized as risk factors for cardiovascular disease. Although whether dyslipidemia increases the risk for AD remains unclear, dyslipidemia as a vascular risk factor could be involved in dementia through vascular diseases including metabolic syndrome^45^. To explain the mechanism leading to the alteration in VEGF and sVEGFR levels in patients with AD, we investigated the association between VEGF and lipid. Oxidized LDL is associated with VEGF induction, while hyperlipidemia is associated with serum VEGF levels^46,47^. There is a positive correlation between the levels of triglycerides (TG) and sVEGFR2 in patients with diabetes^17^. Our study demonstrates that sVEGFR1 levels are negatively associated with total cholesterol (TC) and LDL. However, there was no association between VEGF and lipid parameters. Under conditions of high LDL (>130 mg/dl) or high TC (>220 mg/dl), VEGF, VEGF/sVEGFR1, and VEGF/sVEGFR2 levels were significantly increased in patients with AD compared with control subjects. Under normal conditions, however, neither VEGF nor sVEGFR2 levels in the blood were altered in patients with AD compared with control subjects. These results indicate that VEGF and sVEGFR might be associated with dysregulated lipid conditions in AD.

In this study, we found that VEGFR2 and sVEGFR2 levels are significantly decreased in Aβ-treated endothelial cells which might implicate decreased plasma sVEGFR2 levels in AD. VEGFR2 protein levels are significantly decreased in transgenic mouse brain tissue. Decreased sVEGFR2 and VEGFR2 might be important to understand the correlation with dysregulation of VEGF signaling in AD.

In conclusion, our results indicate that plasma sVEGFR1 and sVEGFR2 levels are significantly decreased in patients with AD. We analyzed the correlations between plasma VEGF, sVEGFR1, sVEGFR2 levels and lipid parameters that might be involved in dysregulated lipid conditions in AD. We suggest that the alterations of VEGF and its two receptors levels might be associated with those at risk for Alzheimer’s disease.

## Methods

### Subjects

The control, mild cognitive impairment (MCI) and dementia subjects were selected from the population-based Ansan Geriatric (AGE) cohort established in 2002 to study common geriatric diseases of elderly Koreans aged 60 to 84 years. The sampling protocol and design of the AGE Study have been previously described^48,49^. Cognitive functioning and memory impairments of subjects were assessed using a Korean version of Consortium to Establish a Registry for Alzheimer’s disease (CERAD-K) neuropsychological battery^50^. The basic structures of all measures in the original CERAD batteries were maintained in Korean translation. All participants were clinically evaluated according to published guidelines, and each dementia patient met the criteria for the Diagnostic and Statistical Manual of Mental Disorders, fourth edition^51^. All dementia patients met the criteria for probable AD established by the National Institute of Neurological and Communicative Disorders and Stroke and the Alzheimer’s Disease and Related Disorders Association (NINCDS–ADRDA)^49^. MCI was diagnosed on the basis of the Mayo Clinic criteria^52^ as described previously^53,54^. In total, blood samples from 271 subjects were included, and the distribution of control, MCI, dementia subjects are shown in Table 1. The study subjects consisted of 76 dementia patients (average age 75.1 ± 0.6, 18 males, 58 females), 75 subjects with MCI (average age 73.01 ± 0.51, 29 males, 46 females), and 120 unrelated healthy control subjects (average age 71.9 ± 0.42, 50 males, 70 females). Table 1 summarizes demographic and clinical measures for all covariates tested here, including diagnosis (normal, MCI, dementia), the mini-mental state exam (MMSE), and global clinical dementia rating (CDR). CDR scores are 0 for normal, 0.5 for questionable dementia, 1 for mild dementia, 2 and 3 for moderate to severe dementia^55^. All participants provided written informed consent and the study has been approved by the Institutional Review Board (IRB) of the Korea Centers for Disease Control and Prevention (KCDC). All experiments were performed in accordance with relevant guidelines and regulations.

### Animals

APPsw/PS1ΔE9 transgenic mice were used for this study, as previously reported^56^. All experimental protocols were performed in compliance with the guidelines for the care and use of laboratory animals by the Korea Centers for Disease Control and Prevention (KCDC) and approved by the Institutional Animal Care and Use Committee (IACUC) of the KCDC.

### Cell cultures

Human umbilical vein endothelial cells (HUVECs) (Lonza, Walkersville, MD, USA) were cultured in Endothelial Growth Medium-2 (EGM-2)-MV BulletKit (Lonza) with 2% fetal bovine serum (FBS) at 37% in a humidified incubator with 5% CO_2_, as previously described passages^57^ 6–9 were used for experimentation. Primary human brain microvascular endothelial cells (HBMECs) were from Cell systems (Kirkland, WA, USA) and maintained in CSC complete medium with 10% serum and CultureBoost (Cell systems). All primary HBMECs cultures were used between passage 4 and 9. Human iPSC-derived neural progenitor stem cells were obtained from Axol Bioscience (Little Chesterford, UK) and were differentiated to cerebral cortical neurons in approximately 7 days following the recommended manufacturer’s protocol.

### Antibodies and Reagents

The following primary antibodies were used: anti-VEGFR2 (Cell Signaling Technology, MA, USA, 9698S), anti-VEGFR1 (GeneTex, CA, USA, GTX61100), anti-Actin (Millipore Corporation, MA, USA, MAB1501). Specific antibody to splicing variant form of VEGFR2 (sVEGFR2) was purchased from Acris Antibodies (Herford, Germany, AP26034PU-L). Amyloid-beta peptides 1–40 (Aβ_1–40_) were purchased from Invitrogen (CA, USA) and dissolved in hexafluoreisopropanol (HFIP) for 2 h at room temperature, and lyophilized peptide was dissolved in dimethylsulfoxide (DMSO).

### Transient transfection

Cells were transiently transfected with the human Notch intracellular domain (NICD) cloned into the IRES-eGFP vector. The Lipofectamine2000 reagent and Opti-MEM medium (Life technology, NY, USA) were used to transfection according to the manufacturer’s instructions. After 48 h of incubation, cells were harvested for total RNA isolation.

### ELISA measurements

All the cell-free plasma samples were stored in aliquots at −80 °C until assayed collectively by an investigator who was blinded to patient assignment. Enzyme-linked immunosorbent assays (ELISA) were used to measure the levels of VEGF-A, VEGFR1, VEGFR2 according to the manufacturer’s instructions (R&D systems, MN, USA). The ELISA kits detect each secreted forms of VEGF-A, VEGFR1, and VEGFR2 in human plasma.

### Western blotting

Cells and mouse cortex regions were collected and homogenized in radio-immunoprecipitation assay buffer (RIPA buffer; 20 mM Tris, pH 7.4, 150 mM NaCl, 1 mM Na_3_VO_4_, 10 mM NaF, 1 mM EDTA, 1 mM EGTA, 0.2 mM PMSF, 1% Triton X-100, 0.1% SDS, 0.5% deoxycholate), protein concentrations were determined using a Bradford protein assay following the manufacturer’s instruction^58^. Bolt 4 ~ 12% Bis-Tris gradient gels were used for SDS-PAGE in MES SDS buffer (Life technology, NY, USA). The primary antibodies were diluted in PBS with 5% nonfat dry milk and 0.1% Tween 20 as follows: anti-VEGFR1 (1:1,000), anti-VEGFR2 (1:1,000), anti-sVEGFR2 (1:1,000), and anti-Actin (1:10,000).

### Real-time reverse transcription polymerase chain reaction

Real-time quantitative RT-PCR analysis was performed using SYBR Green PCR core reagent, in two-step RT-PCR protocol according to the manufacturer’s protocol (Applied Biosystems, Warrington, UK). Initial denaturation at 95 °C for 10 min was followed by 40 amplification cycles of 95 °C for 15 seconds and 58 °C for 1 min. The primer sequences for the RT-PCR experiments were as follows; VEGF-A sense 5′-CACACCCACCCACATACATA-3′ and antisense 5′-CAACTCAAGTCCACAGCAGT-3′; sVEGFR1 sense 5′-ACAATCAGAGGTGAGCACTGCAA-3′ and antisense 5′-TCCGAGCCTGAAGTTAGCAA -3′; sVEGFR2 sense 5′-TTCTTGGCTGTGCAAAAGTG-3′ and antisense 5′-TCTTCAGTTCCCCTCCATTG-3′; VEGFR1 sense 5′-GACAAATCCTGACTTGTACCGC-3′ and antisense 5′-TGCTCTCAATTCTGTTTCCCAT-3′; VEGFR2 sense 5′-GAGAGTTGCCCACACCTGTT-3′ and antisense 5′-CAACTGCCTCTGCACAATGA-3′; NOTCH-1 sense 5′-GAGGCGTGGCAGACTATGC-3′ and antisense 5′-CTTGTACTCCGTCAGCGTGA-3′; GAPDH sense 5′-CAGCCTCAAGATCATCAGCA-3′ and antisense 5′-TGTGGTCATGAGTCCTTCCA-3′. The relative quantification was normalized to the GAPDH gene expression levels. PCR reactions were performed using ABI Prism 7900 SDS (Applied Biosystems, Warrington, UK). The mean threshold cycle (Ct, the first cycle at which an exponential growth of PCR product is detected) value of stimulated sample was compared to that of unstimulated control sample using the Ct value of GAPDH as an internal control. ΔCt was the difference in Ct values derived from each gene (in each sample assayed) and GAPDH gene, while ΔΔCt represented the difference between paired samples. All experiments were performed as triplicated.

### Statistical analyses

Data were expressed as mean ± standard error of the mean (SEM). To analyze demographic and plasma levels of target proteins between dementia, MCI and control groups, Kruskal-Wallis test was performed followed by Mann-Whitney U-tests. Correlation between factors was analyzed by Spearman’s method. Statistical analyses were performed using SPSS 12.0 (IBM, NY, USA). Values of *p* < 0.05 was considered statistically significant.

## Electronic supplementary material

Supplementary Information
