# Supplementary material for: VEGFR2 alteration in Alzheimer’s disease
Source: Sci Rep. 2017 Dec 18;7:17713. doi: 10.1038/s41598-017-18042-1 (PMC5735090; doi:10.1038/s41598-017-18042-1)
Supplement: Supplementary file 1 — Supplementary Information [file 41598_2017_18042_MOESM1_ESM.doc]

**Supplementary Information**

**VEGFR2 alteration in Alzheimer′s disease**

Sun-Jung Cho^1^, Moon Ho Park^2^, Changsu Han^3^, Keejung Yoon^4^ and Young Ho Koh^1^

^1^ Division of Brain Diseases, Center for Biomedical Sciences, Korea National Institute of Health, 187 Osongsaengmyeong2-ro, Osong-eup, Heungdeok-gu, Cheongju-si, Chungcheongbuk-do 28159, Korea. Departments of ^2^ Neurology and ^3^ Psychiatry, Korea University Medical College, Ansan Hospital, 123 Jeokgeum-ro, Danwon-gu, Ansan-si, Gyeonggi-do 15355, Korea. ^4^College of Biotechnology and Bioengineering, Sungkyunkwan University, 2066 Seobu-ro, Jangan-gu, Suwon-si, Gyeonggi-do 16419, Korea.

*Correspondence and requests for materials should be addressed to Y.H.K (email: [kohyoungho122@gmail.com](mailto:kohyoungho122@gmail.com)).

**Supplementary Table 1. Correlation between plasma biomarkers and clinical rating scales**

| Features | sVEGFR2 | | sVEGFR1 | | VEGF | | VEGF/sVEGFR2 | | VEGF/sVEGFR1 | |
| --- | --- | --- | --- | --- | --- | --- | --- | --- | --- | --- |
|  | rho | *p*-value | rho | *p*-value | rho | *p*-value | rho | *p*-value | rho | *p*-value |
| Ages | -0.15 | **0.01** | -0.002 | 0.97 | 0.001 | 0.9 | 0.04 | 0.43 | 0.003 | 0.95 |
| MMSE | 0.69 | 0.25 | 0.139 | **0.02** | -0.187 | **0.002** | -0.2 | **0.001** | -0.21 | **<0.001** |
| CDR | -0.172 | **0.004** | -0.09 | 0.14 | 0.11 | 0.067 | 0.16 | **0.007** | 0.115 | 0.058 |
| sVEGFR2 | - | **-** | 0.154 | **0.011** | -0.07 | 0.25 | -0.4 | **<0.001** | -0.103 | 0.09 |
| sVEGFR1 | 0.154 | **0.011** | - | - | -0.156 | **0.01** | -0.18 | **0.003** | -0.473 | **<0.001** |
| TC | 0.102 | 0.94 | -0.187 | **0.002** | -0.011 | 0.85 | -0.047 | 0.43 | 0.05 | 0.4 |
| TG | 0.13 | **0.03** | -0.064 | 0.295 | -0.018 | 0.77 | -0.053 | 0.38 | 0.015 | 0.8 |
| HDL | 0.22 | 0.72 | 0.016 | 0.792 | 0.02 | 0.74 | 0.005 | 0.92 | 0.012 | 0.837 |
| LDL | 0.027 | 0.65 | -0.164 | **0.007** | 0.06 | 0.92 | -0.0065 | 0.91 | 0.052 | 0.38 |

Spearman rank correlation coefficient test was used for assessment of correlation.

**Supplementary Table 2. Correlation between plasma biomarkers and lipid parameters**

| Features | | sVEGFR2 | | sVEGFR1 | | VEGF | | VEGF/VEGFR2 | | VEGF/VEGR1 | |
| --- | --- | --- | --- | --- | --- | --- | --- | --- | --- | --- | --- |
|  |  | rho | *p*-value | rho | *p*-value | rho | *p*-value | rho | *p*-value | rho | *p*-value |
| TC | control | 0.174 | 0.058 | -0.139 | 0.131 | -0.099 | 0.28 | -0.15 | 0.12 | -0.05 | 0.58 |
|  | MCI | 0.039 | 0.738 | -0.232 | **0.045** | -0.025 | 0.833 | -0.026 | 0.82 | 0.093 | 0.42 |
|  | Dementia | 0.086 | 0.459 | -0.154 | 0.184 | 0.104 | 0.37 | 0.05 | 0.66 | 0.138 | 0.23 |
|  |  |  |  |  |  |  |  |  |  |  |  |
| TG | control | 0.14 | 0.126 | -0.01 | 0.91 | -0.097 | 0.294 | -0.116 | 0.2 | -0.074 | 0.42 |
|  | MCI | -0.067 | 0.565 | -0.017 | 0.882 | -0.019 | 0.87 | 0.009 | 0.94 | 0.028 | 0.81 |
|  | Dementia | 0.406 | **0.0002** | -0.165 | 0.155 | 0.024 | 0.84 | -0.128 | 0.27 | 0.06 | 0.57 |
|  |  |  |  |  |  |  |  |  |  |  |  |
| LDL | control | 0.11 | 0.22 | -0.144 | 0.116 | -0.112 | 0.223 | -0.147 | 0.108 | -0.062 | 0.501 |
|  | MCI | 0.005 | 0.968 | -0.259 | **0.025** | 0.011 | 0.923 | 0.18 | 0.87 | 0.111 | 0.343 |
|  | Dementia | -0.062 | 0.595 | -0.046 | 0.693 | 0.151 | 0.194 | 0.152 | 0.18 | 0.146 | 0.208 |

**Supplementary Table 3. Analysis of sVEGFR2, sVEGFR1, VEGF, and VEGF/sVEGFR2 VEGF/sVEGFR1 with levels of LDL in plasma**

|  |  |  |  |  |
| --- | --- | --- | --- | --- |
| Features | Normal Control | Amnestic MCI | Dementia | *p*-value |
| N | 120 | 75 | 76 |  |
| LDL>130 mg/dl | 57(48%) | 23(31%) | 33(43%) |  |
|  |  |  |  |  |
| **sVEGFR2 (ng/ml)** |  |  |  |  |
| LDL<130 mg/dl | 7.28±0.18 | 7.25±0.18 | 6.9±0.19 | 0.42 |
| LDL>130 mg/dl | 7.52±0.15 | 7.04±0.16 | 6.8±0.19^a^ | **0.02** |
| **sVEGFR1 (pg/ml)** |  |  |  |  |
| LDL<130 mg/dl | 126.7±3.4 | 119±3.8 | 107.2±2.5 | **0.023** |
| LDL>130 mg/dl | 120.1±7.2 | 111±7.9 | 107.4±2.6 | 0.349 |
| **VEGF (pg/ml)** |  |  |  |  |
| LDL<130 mg/dl | 149.2±12.1 | 189±26.8 | 157±10.6 | 0.341 |
| LDL>130 mg/dl | 134.5±8.4 | 175±32.9 | 181±23.5^b^ | 0.064 |
| **VEGF/sVEGFR2** |  |  |  |  |
| LDL<130 mg/dl | 20.8±1.5 | 20.7±4.2 | 23.6±1.7 | 0.338 |
| LDL>130 mg/dl | 18.3±1.2 | 24.8±4.4 | 27.4±3.6^c^ | **0.01** |
| **VEGF/sVEGFR1** |  |  |  |  |
| LDL<130 mg/dl | 1.29±0.1 | 1.71±0.26 | 1.51±0.11 | 0.206 |
| LDL>130 mg/dl | 1.24±0.08 | 1.72±0.38 | 1.76±0.24^d^ | 0.074 |
|  | | | |  |

^a^compared with control; *p* = 0.013

^b^compared with control; *p* = 0.019

^c^compared with control; *p* = 0.003

^d^compared with control; *p* = 0.025

**Supplementary Table 4. Analysis of sVEGFR2, sVEGFR1, VEGF, and VEGF/sVEGFR2 VEGF/sVEGFR1 with levels of TC in plasma**

|  |  |  |  |  |
| --- | --- | --- | --- | --- |
| Features | Normal Control | Amnestic MCI | Dementia | *p*-value |
| N | 120 | 75 | 76 |  |
| TC(>220) | 23(19%) | 17(23%) | 22(29%) |  |
|  |  |  |  |  |
| **sVEGFR2 (ng/ml)** |  |  |  |  |
| Low TC | 7.34±0.14 | 7.17±0.16 | 6.83±0.16 | 0.084 |
| High TC(>220) | 7.63±0.21 | 7.22±0.25 | 7.28±0.18 | 0.245 |
| **sVEGFR1 (pg/ml)** |  |  |  |  |
| Low TC | 121.5±5.6 | 118±3.6 | 108.9±2.4 | 0.123 |
| High TC(>220) | 110.1±2.9 | 113.2±10.5 | 103.1±2.0 | 0.227 |
| **VEGF (pg/ml)** |  |  |  |  |
| Low TC | 148.1±8.8 | 181.4±24.2 | 154.5±9.7 | 0.555 |
| High TC(>220) | 117.5±11.4 | 197.6±43.2 | 200±32.8^a^ | **0.002** |
| **VEGF/ sVEGFR2** |  |  |  |  |
| Low TC | 20.6±1.1 | 26.6±3.8 | 23.5±1.6 | 0.271 |
| High TC(>220) | 15.6±1.5 | 27.6±5.8 | 29.7±4.9^b^ | **0.001** |
| **VEGF/ sVEGFR1** |  |  |  |  |
| Low TC | 1.31±0.08 | 1.64±0.23 | 1.48±0.10 | 0.467 |
| High TC(>220) | 1.07±0.1 | 1.94±0.51 | 1.97±0.33^c^ | **0.001** |
|  | | | |  |

^a^compared with control; *p* = 0.0007

^b^compared with control; *p* = 0.0002

^c^compared with control; *p* = 0.0002

**Supplementary Table 5. Correlation between ApoE ε4 carriers and sVEGFR2 levels**

|  |  |  |  |  |
| --- | --- | --- | --- | --- |
| Features | Normal Control | Amnestic MCI | Dementia | *p*-value |
| N | 120 | 75 | 76 |  |
| ApoE ε4 allele, N(%) |  |  |  |  |
| negative (-) | 9780%) | 66(88%) | 50(65.8%) |  |
| positive (+) | 23(20%) | 9(12%) | 26(34.2%) |  |
|  |  |  |  |  |
| **sVEGFR2 (ng/ml)** |  |  |  |  |
| negative (-) | 7.34± 0.13 | 7.14± 0.14 | 7.06± 0.17 | 0.456 |
| positive (+) | 7.5 ± 0.24 | 7.5± 0.5 | 6.57± 0.22^a^ | **0.025** |
| **sVEGFR1 (pg/ml)** |  |  |  |  |
| negative (-) | 121.5± 5.5 | 117.4± 3.9 | 105.9± 2.3 | **0.006** |
| positive (+) | 109.1± 4.4 | 113.3± 8.9 | 109.8± 3.1 | 0.852 |
| **VEGF (pg/ml)** |  |  |  |  |
| negative (-) | 140.7± 8.5 | 166.1± 15.1 | 174± 16.2 | **0.05** |
| positive (+) | 147.3± 14.7 | 324.4± 132 | 155.4± 15.1 | 0.421 |
| **VEGF/sVEGFR2** |  |  |  |  |
| negative (-) | 19.5± 1.12 | 24.1± 2.3 | 25.5± 2.4 | **0.037** |
| positive (+) | 20.4± 2.3 | 47.2± 20.7 | 24.8± 3.0 | 0.247 |
| **VEGF/sVEGFR1** |  |  |  |  |
| negative (-) | 1.23± 0.07 | 1.54± 0.17 | 1.69±0.17 | **0.007** |
| positive (+) | 1.43± 0.16 | 2.96± 1.23 | 1.48± 0.17 | 0.501 |
|  | | | |  |

^a^compared with control; *p* = 0.009

**Supplementary Figures**

**Supplementary Figure 1. Full blot of Fig. 2d**


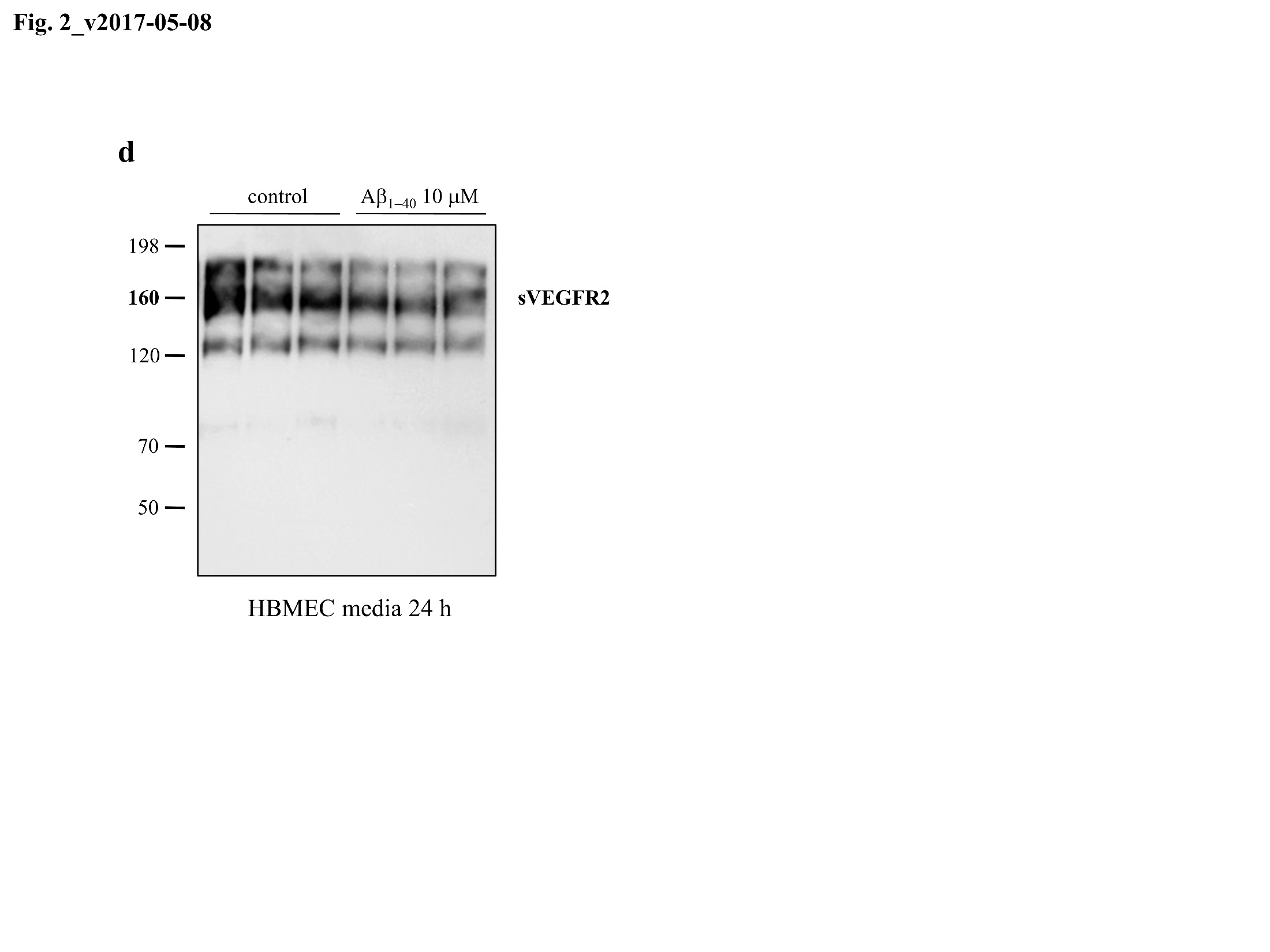


**Supplementary Figure 2. Full blots of Fig. 3a, 3b, and 3c**


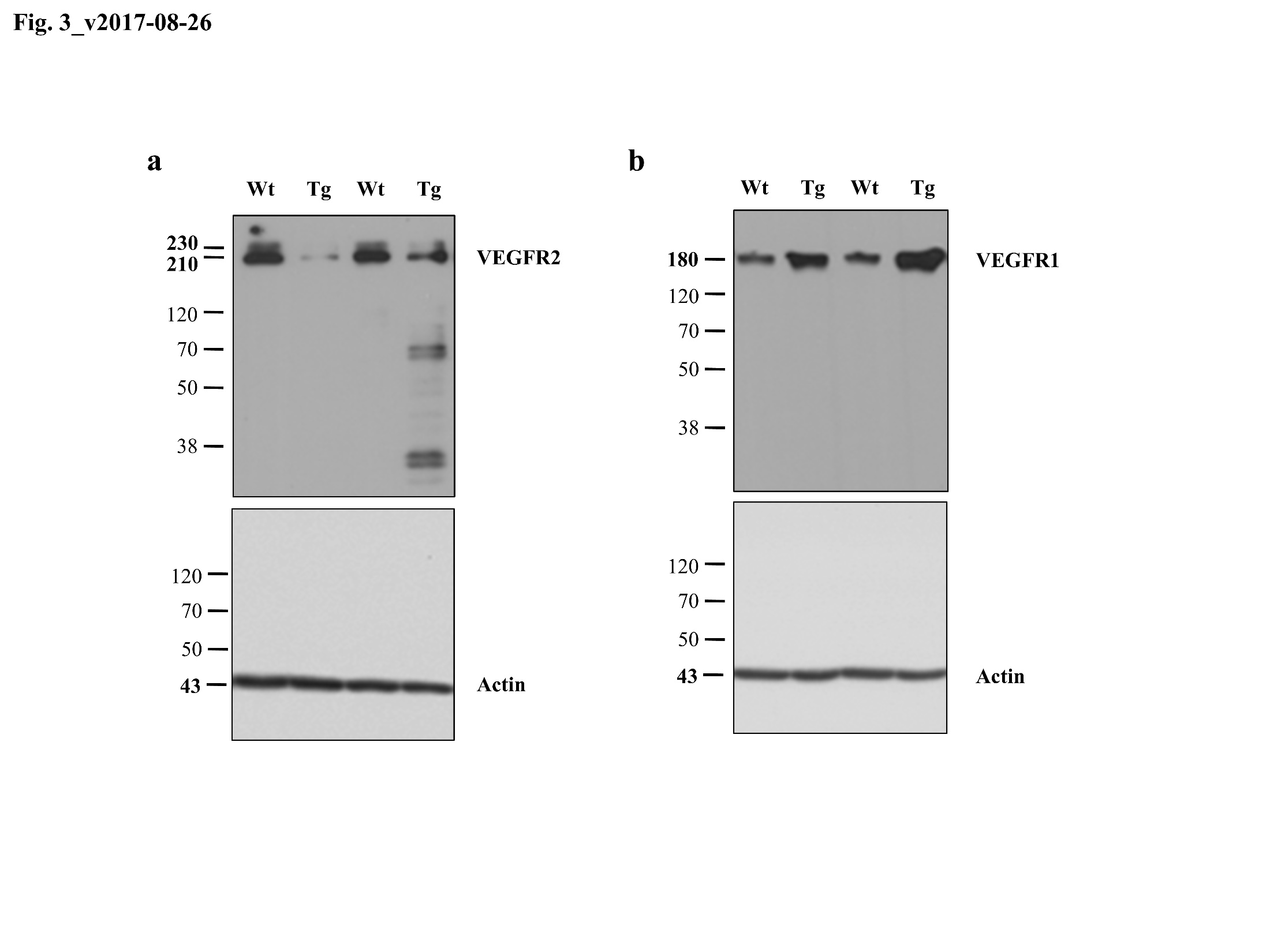


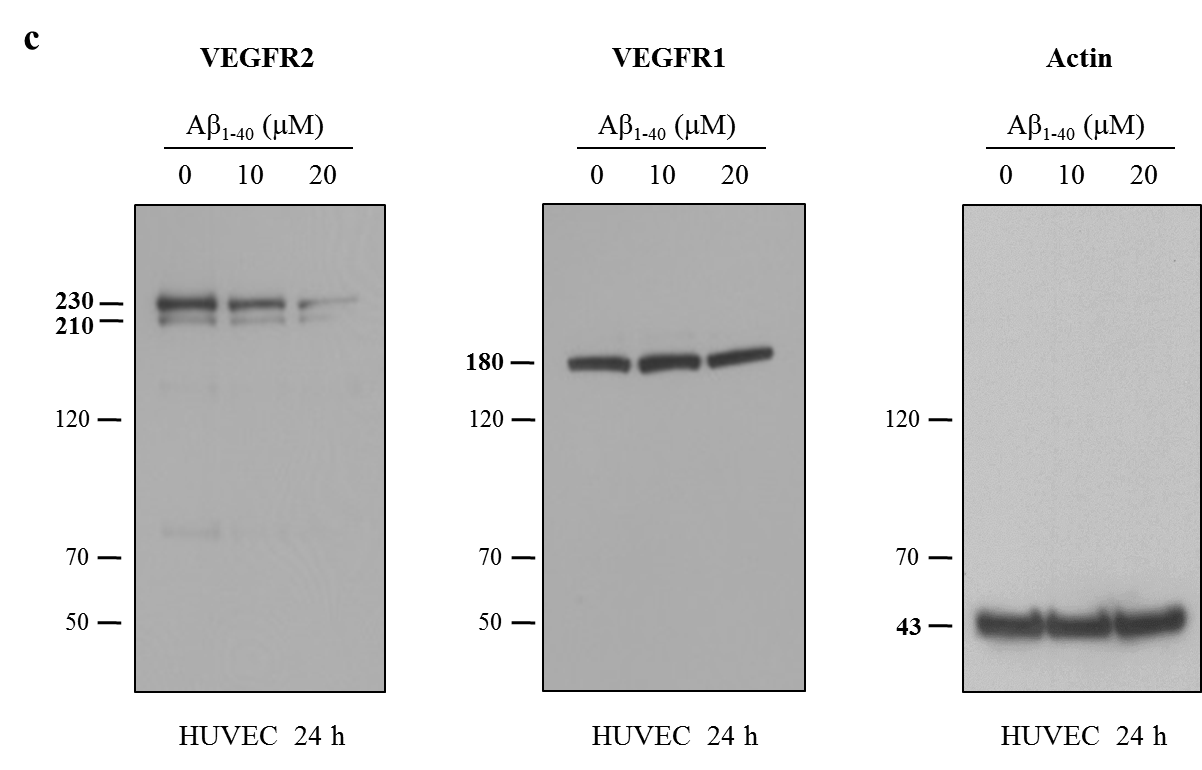


**Supplementary Figure 3. GFP fluorescent images show the transient transfection efficiency of Fig. 4b**

**
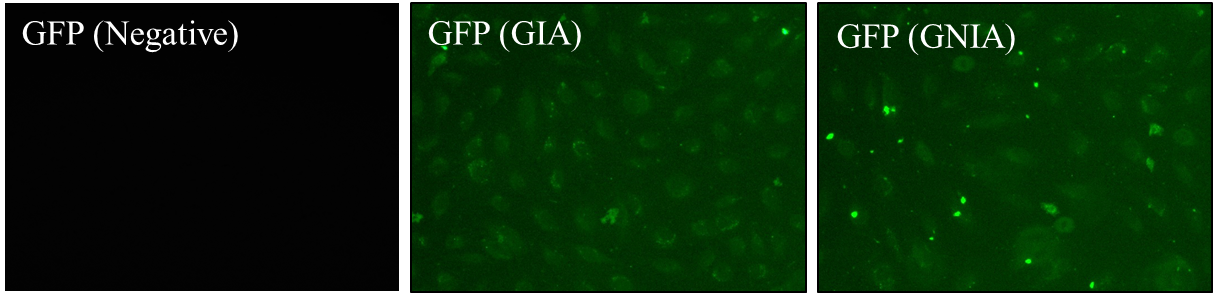
**

**Supplementary Figure 3. GFP expressions in transiently transfected HUVEC.** HUVEC were transiently transfected with the control GFP (GIA), GFP-linked Notch intracellular domain (GNIA) cloned into the IRES-eGFP vector, and un-transfected as negative control (Negative). After 48 h of incubation, fluorescent images show GFP expressions in HUVEC.
